# An Overview on Recent Progress in Electrochemical Biosensors for Antimicrobial Drug Residues in Animal-Derived Food

**DOI:** 10.3390/s17091947

**Published:** 2017-08-24

**Authors:** Marjan Majdinasab, Mustansara Yaqub, Abdur Rahim, Gaelle Catanante, Akhtar Hayat, Jean Louis Marty

**Affiliations:** 1 Department of Food Science & Technology, Shiraz University, Shiraz 71441-65186, Iran; majdinasab@shirazu.ac.ir; 2 Interdisciplinary Research Centre in Biomedical Materials (IRCBM), COMSATS Institute of Information Technology, Lahore 54000, Pakistan; mustansarayaqub@ciitlahore.edu.pk (M.Y.); abdurrahim@ciitlahore.edu.pk (A.R.); 3 BAE: Biocapteurs-Analyses-Environnement, Universite de Perpignan Via Domitia, 52 Avenue Paul Alduy, Perpignan CEDEX 66860, France; gaelle.catanante@univ-perp.fr

**Keywords:** electrochemical biosensors, antimicrobial drug residue, food safety, kanamycin, chloramphenicol, tetracycline, streptomycin

## Abstract

Anti-microbial drugs are widely employed for the treatment and cure of diseases in animals, promotion of animal growth, and feed efficiency. However, the scientific literature has indicated the possible presence of antimicrobial drug residues in animal-derived food, making it one of the key public concerns for food safety. Therefore, it is highly desirable to design fast and accurate methodologies to monitor antimicrobial drug residues in animal-derived food. Legislation is in place in many countries to ensure antimicrobial drug residue quantities are less than the maximum residue limits (MRL) defined on the basis of food safety. In this context, the recent years have witnessed a special interest in the field of electrochemical biosensors for food safety, based on their unique analytical features. This review article is focused on the recent progress in the domain of electrochemical biosensors to monitor antimicrobial drug residues in animal-derived food.

## 1. Introduction

Human health is greatly influenced by environment, particularly the quality and nature of the food consumed. Therefore, in recent decades, food safety and its quality have become prime concerns owing to the growing food consumption and rapidly changing dietary habits. According to a survey, unhealthy and contaminated food is associated with approximately 2 million deaths annually all over the world. In response, public health agencies all over the world are working to put forward stringent safety measures and food regulations [1,2].

The various antibiotic families used so far in veterinary medicines include sulphonamides, lincosamides, nitrofurans, trimethoprim, amphenicols, tetracyclines, polymyxins and β-lactams and quinolones [3,4,5]. Residues of these drug can pose serious health hazards by contaminating food products consumed by humans such as milk, chicken, egg, honey, fish or meat [6]. The drug residues in animal origin food can also trigger the development of antimicrobial resistance, which has become a serious international issue in recent years [7,8]. These resistant bacterial pathogens can be transferred to human beings through the food chain which may lead to the inefficiency of antibiotic therapy in humans infected with resistant pathogens resulting in a rise in morbidity and mortality rates.

### Legislations

Drug residues, as defined by the European Union (EU) and the Center for Veterinary Medicine, an agency under the Food and Drug Administration (FDA/CVM) in the USA are “pharmacologically active substances (whether active principles, recipients or degradation products) and their metabolites which remain in foodstuffs obtained from animals to which the veterinary medicines in question has been administered” [9]. To reduce the adverse effects and toxicity of antibiotic-resistant pathogens, the European Union has put forward a “precautionary principle” model whereby some specific antimicrobial growth promoters are banned [10], while for the veterinary medicines that are not banned, maximum residue limits (MRLs) have been established by a number of regulatory authorities in the United States and European countries to ensure consumers’ safety from exposure to harmful drug residues in animal origin food. The European Union has defined MRL as “the maximum legally acceptable quantity of pharmacologically active substances (whether active principles, excipients or metabolites) and their degradation products in food products derived from animals”. Usually, MRL is adjusted according to the acceptable daily intake (ADI), which also incorporates a maximum safety margin in its calculation. These calculations are also dependent upon the withdrawal period. The withdrawal period is the time elapsed between last dosage of a certain drug or any pharmacologically active substance administered to the individual and the time at which the level of drug residue in food products i.e., meat, eggs, milk or in animal tissues i.e., liver, muscles, is equal to or less than MRL of that particular antibiotic drug. For instance, the Canadian MRLs for streptomycin, penicillin, sulfonamides and tetracycline have been calculated to be 0.5 ppm (chicken muscle), 0.05 ppm (chicken muscle), 0.1 ppm (cattle muscle and 0.2 ppm (chicken muscle) respectively [11].

Keeping in mind the above facts, it is the need of the day to develop reliable screening methods for selective and sensitive monitoring of veterinary drug residue levels in animal derived food to ensure the safe and quality food supply and to curtail their effects on consumers’ health. To date several analytical methods have been developed to determine the drug residue levels in food products originated from animals. Usually these methods can be divided into two groups, namely, screening and confirmatory methods. Screening methods usually provides qualitative or semi-quantitative results about the analyte. Screening approaches are considered a viable approach owing to their easy handling, very low margin of false positive results, good selectivity and cost effectiveness [12]. Once a screening method gives a positive result, the next step will be the implementation of a confirmatory method. Confirmatory approaches are mostly based on liquid chromatography coupled with mass spectroscopy (LC-MS), gas chromatography/mass spectrometery (GC-MS), and high performance liquid chromatography (HPLC) [13]. Although biosensor technology is not well established for confirmatory tests, however, these can provide a future tool for screening purposes in the domain of antimicrobial drug residue monitoring.

## 2. Biosensors as an Alternative Analytical Tool

Bioanalysis has been carried out by human beings forever, with the use of the nerve cells of the nose to detect scents or the enzymatic reactions on the tongue to taste food. With progress in understanding about the function of living organisms, scientific research has integrated them into man-made reactions to detect trace amounts of biochemicals in complex systems. Using bioreceptors from biological organisms or receptors, biosensors have been employed as a new mean of analytical and chemical analysis. The field of biosensors originated from the work by Clark and Lyons [14], Guilbault et al. [15], Updike and Hicks [16] and Guilbault and Montalvo [17]. Later on, a mediated electrochemical biosensor using ferrocene to detect electroactive species was described by Di Gleria et al. [18]. This work led to the successful commercialization of a glucose pen by Medisens. Since these first reports, there has been an extensive growth in the field of biosensors with a wide variety of applications in biological monitoring and environmental sensing [19].

**Figure 1:**
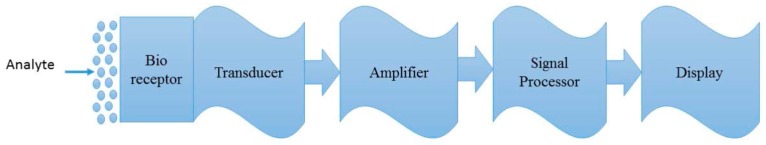
Schematic diagram of a biosensor.

According to The National Research Council (part of the US National Academy of Science), a biosensor is defined as a detection device that incorporates a living organism or product derived from a living system as the recognition element or a bioreceptor and a transducer to convert a biological reaction into a measurable signal or indication (Figure 1).

The most important properties of a biosensor such as specificity and sensitivity depend on the bioreceptor, biomolecule immobilization method and transduction method/surface. The high specificity of the system can be successfully achieved only if there is a strong efficient coupling reaction between the biological and transducer components. Bioreceptors are the key factors in the fabrication of biosensor and can take many forms. Numerous biorecognition elements have been used in biosensing, however, they can be classified into the following major categories:

1. Enzymes which catalyse specific biochemical reactions
2. Antibodies known as immunoglobulins which form an important part of a biological group termed binding proteins and bind a particular substance with high affinity
3. RNA/DNA aptamers are ligands selected to have high binding affinity and specificity to a target molecule
4. Synthetic molecularly imprinted polymers to replace biomolecules
5. Bacteria (genetically modified or not)

For veterinary drug residue detection, the most frequently used biosensors are those based on antibody/antigen affinity pairs, which are widely used in the immunochemical screening of samples. These affinity assays mainly make use of optical transducer systems, however, there are also reports on electrochemical-based output signals in the literature.

Electrochemical biosensors find widespread application in a diversified area, such as food quality control, environmental monitoring, and clinical analysis [20]. Recently, studies in this area emphasize novel sensing strategies with specific consideration of the augmentation of specificity, response time, and sensitivity. The electrochemical transducer selectivity can be enhanced with chemical or electrochemical modifications. Mostly, the electrode surface can be modified with the immobilization of reagents or an electrochemical pre-treatment which enhances the electrochemical properties of the bare electrode surface [21]. Furthermore, the electrochemical biosensor sensitivity is greatly influenced by the application of transduction principles. Depending on the electrochemical principle involved, sensors can be categorized as potentiometric, amperometric, voltammetric, impedimetric and conductivity sensors [22]. An amperometric biosensor measures the current produced when an electroactive species is oxidized or reduced at a bioreceptor-coated (or antigen-coated) electrode to which an analyte (or bioreceptor) binds specifically. Potentiometric devices measure the changes in pH and ion concentration when a biorecognition element immobilized on the electrode surface interacts with an antigen in the sample. The potential difference between the electrode modified with the biorecognition element and a reference electrode is a function of the concentration of analyte in the sample. Conductimetric biosensors quantify the electrical conductivity changes in a solution at constant voltage, generated by biochemical reactions which particularly produce or consume ions. This type of transducer has a limited response for the detection of antibiotics due to its poor signal/noise ratio [23,24]. Electrochemical biosensors have advantages, such as their simplicity, rapidity, portability ease of fabrication, field applicability and low cost, making them attractive sensing devices for use as alternative analytical methods, with accurate and fast responses without sample pre-treatment, opening the possibility of direct on-site analysis with intuitive devices [25].

In the same context, aptamers have attracted great interest in the field of electrochemical biosensing. Apart from having the same or even higher sensitivity and selectivity as compared to antibodies, aptamers offer the advantages of large scale production with less expensive in vitro systems and enhanced environmental stability. Aptamers can be very easily integrated into electrochemical biosensing platforms due to their ease of modification with various functional groups. The probing of the affinity binding event in aptamer-based assays is mainly accomplished with an optical output signal. However, optically-based read out methods of aptamer binding events not only require high precision and expensive instrumentation, but also involve sophisticated numerical algorithms to interpret the data. Alternatively, a number of innovative electrochemical aptasensor designs have been reported in the literature. These type of devices combine aptamers with electrochemical transducers to generate an electrical signal, and provide a simple, accurate and inexpensive platform for diverse types of applications [26,27]. Keeping in mind the abovementioned advantages, it can be clearly seen from the literature that most of the electrochemical biosensors for antimicrobial drug residues reported in the literature are based on the integration of aptamers as biorecognition element.

## 3. Electrochemical Biosensors for Antimicrobial Drug Residues in Animal-Derived Food

Electrochemical biosensors have gained substantial consideration in many areas such as disease diagnosis, food safety, biomedical applications and environmental monitoring [28,29]. Nanomaterials have attracted considerable attention in the field of electrochemical biosensors as a way to achieve better performance, due to their unique electrical and chemical properties. The incorporation of nanomaterials can potentially increase the response speed, selectivity and sensitivity to meet the requirements of detection of contaminants in food samples [30,31]. Numerous nano-materials, such as metal nanomaterials, silica nanoparticles, carbon nanomaterials, and other functionalized nanoparticles, have been used for sensing antimicrobial drug residues in animal-derived food. Biosensors using an aptamer as a recognition element are also commonly used for the detection of antibiotic residues in animal-derived foods. In this context, we highlight the recent progress towards fabrication of electrochemical biosensors to monitor the most frequently employed antimicrobial drugs.

### 3.1. Kanamycin

**Figure 2:**
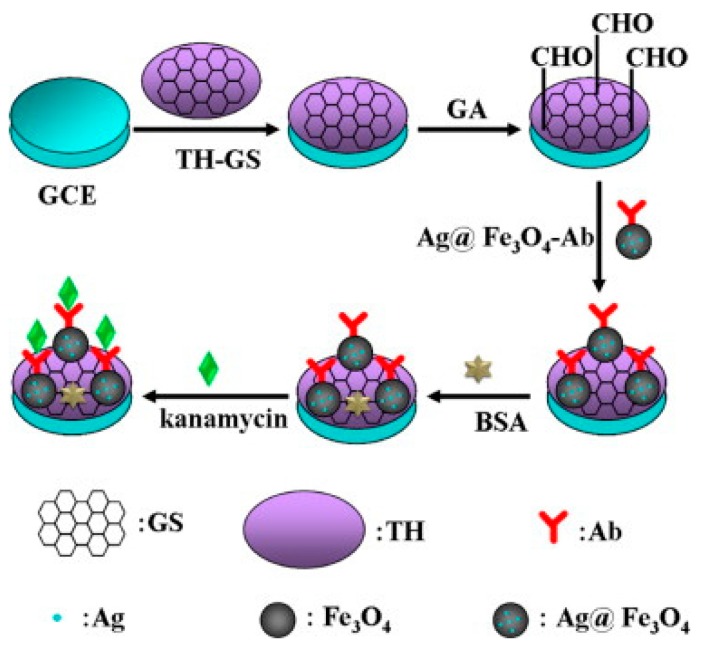
Schematic illustration of the stepwise procedure for the fabrication of a kanamycin immunosensor (reproduced with permission from [37]).

Kanamycin is considered as an important sub-class of aminoglycoside antibiotics produced by the fermentation of *Streptomyces kanamyceticus* [32,33,34]. Keeping in mind health damages, animal-derived foods must be monitored strictly for kanamycin residues. A variety of analytical approaches have been reported for the detection of kanamycin level in contaminated foods and body fluids [35]. A label-free amperometric immunosensor based on graphene sheet-Nafion-thionine-platinum nanoparticles (GS/Nf/TH/Pt)-modified electrode was proposed for the ultrasensitive detection of kanamycin by Qin et al. The proposed immunosensor showed good analytical performance features such as a low detection limit (5.74 pg/mL), wide linear range (from 0.01 to 12.0 ng/mL), high stability, and good selectivity in the detection of kanamycin. The electrochemical immunosensor was employed to monitor kanamycin in various food samples with recovery percentages from 99.4 to 106% [36]. Similarly, a highly sensitive label-free immunosensor for the detection of kanamycin was designed using silver hybridized mesoporous ferroferric oxide nanoparticles (Ag@Fe_3_O_4_ NPs) and thionine-mixed graphene sheet (TH-GS, Figure 2). The proposed immunosensor exhibited excellent performance such as a low detection limit (15 pg mL^−1^), wide linear range (from 0.050 to 16 ng mL^−1^), short analysis time (3 min), high stability, and good selectivity in the detection of kanamycin. The immunosensor was evaluated for pork meat samples [37]. The analytical characteristics of the kanamycin electrochemical immunosensors rein the ported literature are provided in the Table 1 for better understanding of the readers.

Recently aptamer-based biosensors have been drawing attention as efficient analytical tools with good sensitivity [38,39,40,41,42]. Zhu and group reported a label-free aptasensor fabricated by self-assembly of gold (AuNPs)/conducting polymer (2,5-di-(2-thienyl)-1*H*-pyrrol-1-(*p*-benzoic acid)) nano-composite onto a screen printed electrode surface through electropolymerization. The aptamer was anchored to the electrode via covalent linkages between the COOH groups of the aptamer and the −NH_2_ moiety of the polymer. On adding kanamycin, a kanamycin/aptamer conjugate was formed which subsequently produced an enhanced current signal in linear sweep voltammetry. The assay was applied to determine kanamycin with a detection limit of 4.5 ± 0.2 μg/L and recovery percentages of 80.1–98% in food samples [43].

**Figure 3:**
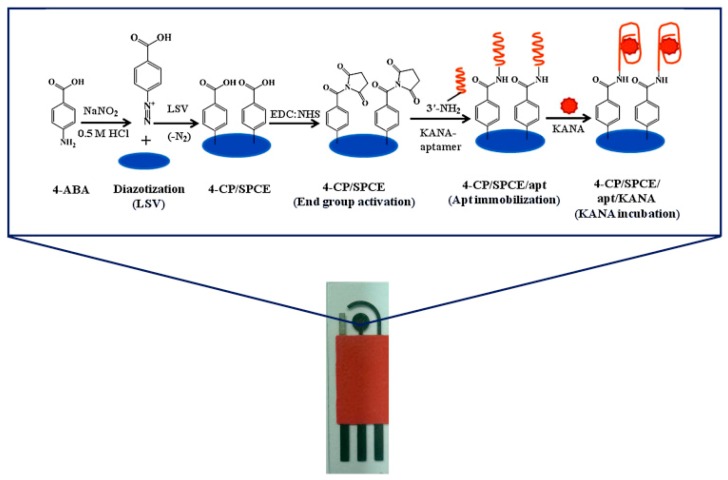
Schematic representation of surface modification and fabrication of an aptasensor for kanamycin (KANA) detection (reprinted with permission from [45]).

In another study, Qin and co-workers developed a label-free aptasensor for kanamycin based on thionine-functionalized graphene. Modified graphene facilitated the charge transfer rate between electrode and analyte thereby offering a wide linear range 5 × 10^−7^–5 × 10^−2^ μg/mL and a detection limit of 0.42 pg/mL. (4-itself). Qin and workers modified a glassy carbon electrode with BMIMPF_6_ ionic liquid and MWCNTs, and subsequently deposited a layer of amino-functionalized graphene to enhance the conductivity of the modified electrode. K-aptamer was immobilized to the electrode surface via phosphoramidate linkages between the aptamer phosphate group and the amino groups of graphene. Differential pulse voltammetry was employed to monitor the electrochemical signals. A reduction in signal intensity was observed with the increased concentration of kanamycin owing to the fact that aptamer/kanamycin complex acted as a barrier to the redox activity at the electrode surface. This electrochemical sensor showed LOD of 0.42 μg/L with a linearity of 0.484–4.845 mg/mL and recovery percentages of 92.15–105.99% [32]. With the aim of proving a portable platform, our group has recently devised a facile, label free and portable aptasensor for the quantitative determination of kanamycin (KANA) by electrochemical impedance spectroscopy (EIS), based on the assembly of in vitro selected single strand DNA (ssDNA) anti-KANA-aptamer-functionalized screen printed carbon electrodes (Figure 3). Under optimized experimental conditions, the devised aptasensor exhibited a dynamic range of 1.2–600 ng mL^−1^ with linearity 1.2–75 ng mL^−1^ and limit of detection of 0.11 ng mL^−1^. For practical applications, the aptasensor performance was verified in spiked milk samples with recovery percentages of 96.88–100.5%. [44]. Table 1 provides insight on the analytical parameters of the electrochemical aptasensors for the detection of kanamycin reported in the literature.

### 3.2. Chloramphenicol

**Figure 4:**
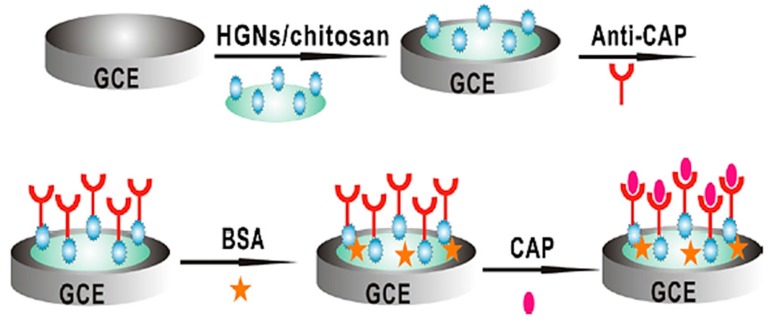
Schematic representation of the electrochemical immunosensor fabrication steps for the detection of chloramphenicol (reproduced from Zhang et al. [54] with permission).

Chloramphenicol (CAP) is a broad-spectrum synthetic antibiotic used for treatment of infectious diseases in humans and animals. High levels of CAP in animal-derived foods have adverse side effects on human health. Among the different detection methods, electrochemical biosensors have attracted more attention towards monitoring of CAP. Yang et al. developed a novel electrochemical sensors based on multiwalled carbon nanotubes@molecularly imprinted polymer (MWCNTs@MIP) for the detection of chloramphenicol [52]. 3-Hexadecyl-1-vinylimidazolium chloride was used as a monomer to prepare MIP on the surface of MWCNTs. Polymerization of functionalized MWCNTs was performed in the presence of chloramphenicol as template. Subsequently, the synthesized MWCNTs@MIP were used for coating a glassy carbon electrode modified with mesoporous carbon (MC) and three-dimensional porous graphene (DPG). The MC and DPG increased the sensitivity of the sensor. The binding of CAP to MWCNTs@MIP was evaluated by cyclic voltammetry and differential pulse voltammetry. The designed immunosensor was used to determine chloramphenicol with a detection limit of 0.032 ng mL^−1^ and linear response ranges of 1.615–161.5 ng mL^−1^ and 161.5–1292 ng mL^−1^. The biosensor performance was validated with spiked milk and honey samples with recovery percentages of 91–104%. The designed biosensor shows a satisfactory LOD with respect to the chloramphenicol MRL (0.3 ng mL^−1^), however, it should also be investigated in a food matrix. Hamidi-Asl et al. designed an electrochemical aptasesnsor for sensitive detection of chloramphenicol, based on gelatin type B as a biocompatible matrix to incorporate CAP aptamers [53]. The mixture of gelatin and CAP aptamers was dropped onto the surface of gold screen printed electrodes. The hydrophilic porous network of gelatin provided an appropriate environment for aptemer entrapment and facilitated the electron transfer between electrode surface and target molecule. Differential pulse voltammetry was employed for the detection of CAP. The designed aptasensor showed a detection limit of 0.059 ng mL^−1^ and a linear range from 0.097 to 0.626 ng mL^−1^. The developed aptasensor was demonstrated with CAP monitoring in spiked skimmed cow’s milk samples with recovery percentages of 82–93%. This biosensor with a simple structure shows a good sensitivity. However, milk samples were spiked with higher concentrations of CAP to evaluate the effect of food matrix on biosensor sensitivity. In another study, a label-free electrochemical biosensor was fabricated for quantification of chloramphenicol [54]. In this electrochemical biosensor, monoclonal antibody was immobilized into hollow gold nanospheres/chitosan composite coated on the surface of glassy carbon electrode (Figure 4). After binding of chloramphenicol to monoclonal antibodies on the surface of electrode, differential pulse voltammetry was used for the detection of CA. Under optimal conditions, a detection limit of 0.06 ng mL^−1^ with a linear range from 0.1 to 1000 ng mL^−1^ was obtained. The designed biosensor was used to determine CAP in meat samples including beef, fish and pork which had been spiked with three concentration levels of CAP (10, 20 and 50 μg g^−1^). The recovery percentages were obtained in the range of 85–93%. However the immunosensor showed a good accuracy in real samples, but the spiking levels were very high compared to the MRL.

Yan et al. fabricated an electrochemical aptasensor for quantification of CAP in honey samples based on target-induced strand release [55]. For the construction of aptasensor, CAP aptamer was coated onto the surface of gold electrode. The immobilized aptamer specifically hybridized with a complementary detection probe. In the absence of CAP, aptamer/detection probe duplex was formed, and a strong electrochemical signal was obtained. In the presence of CAP, aptamer-CAP complex was formed and release of detection probe from the electrode surface resulted in a reduced electrochemical signal. Differential pulse voltammetry was used for the measurement of CAP. Under optimal conditions, the detection limit of the aptasensor was 0.094 ng mL^−1^ with a linear range from 0.323 ng mL^−1^ to 323 ng mL^−1^. The recoveries in honey samples were from 84.4% to 102.0%. The biosensor shows a good sensitivity in buffer solution. The main disadvantage of the designed biosensor is the long assay time where the incubation time with CAP was 2 h and insufficient incubation time could result in incomplete binding for CAP.

Carbon nanomaterials are widely used for the construction of electrochemical biosensors. One of the most attractive carob nanostructures is graphene due to its unique properties such as high surface area, super electrical conductivity and fast electron transfer [56]. Zhang et al. reported the development of an electrochemical sensor for the detection of chloramphenicol using three-dimensional reduced graphene oxide architectures (3DRGOA) [57]. They prepared 3DRGOA through reduction of graphene oxide using zinc foil. The resulted 3DRGOA was coated onto the surface of a glassy carbon electrode (GCE). For detection and quantification of CAP, the interaction between CAP and 3DRGOA/GCE surface was investigated using differential pulse voltammetry. It was observed that the increment in reduction peak current at −633 mV is proportional to the chloramphenicol concentration. The electrochemical sensor was able to detect CAP concentrations as low as 48.45 ng mL^−1^ with a linear range from 323 to 3.65 × 10^4^ ng mL^−1^. According to the LOD results, the designed sensor doesn’t show enough sensitivity when the MRL is set at 0.3 ng mL^−1^. Moreover, the high dilution factor of food samples (300 times) before electrochemical measurement can lead to false negative results considering the low MRL of CAP. The applicability of the designed biosensor for CAP detection was investigated in milk samples and CAP eye drops with recovery percentages of 97.3–104.6%. It is worth mention that 3DRGOA provided a larger surface area and lower resistance towards electron transfer in comparison with conventional two-dimensional graphene for the construction of electrochemical biosensors. The sensor showed a high selectivity, stability, and reproducibility. 

Molybdenum disulfide (MoS_2_) nanosheet is a type of graphene-like two-dimensional nanomaterial which is very attractive in the fabrication of electrochemical sensors/biosensors. Yang et al. investigated the use of molybdenum disulfide nanosheet along with self-doped polyaniline for construction of an electrochemical biosensor [58]. They used ultrasonic exfoliating to incorporate self-doped polyaniline (SPAN) into molybdenum disulfide nanosheets. The resulting nanocomposite provided a highly negative charge surface, due to its negative charges and the benzene rings in the SPAN structure, and was able to adsorb molecules with conjugated structures or positive charges. The obtained nanocomposite was used for coating a carbon paste electrode and then detection of conjugate structured CAP. It was shown that the combination of SPAN and MoS_2_ exhibited a significant synergistic effect to reduce CAP. The designed biosensor detected CAP with a detection limit of 20.99 ng mL^−1^ and linear range of 32.3–3.23 × 10^5^ ng mL^−1^ which is not a satisfactory sensitivity. The biosensor was applied for the measurement of CAP in eye drops and the recovery percentages were 98.2–101.3%. The performance of this biosensor wasn’t checked in food samples.

In another approach, Zheng et al. reported an electrochemical biosensor based on vertical silica mesochannels (VSMs) and cylindrical surfactant micelles (CSMs) for the detection of CAP in milk and honey samples [59]. They modified indium tin oxide (ITO) electrode surfaces using vertical silica mesochannels to support cylindrical surfactant micelles of cetyltrimethylammonium bromide (CTAB) in 2–3 nm channels. CTAB has the property to extract and concentrate organic analytes with lipophilic properties from liquid samples, so the electrode coated with VSMs/CSMs was capable of quantifying CAP with an excellent performance compared to the corresponding uncoated and VSM- coated electrodes. Chloramphenicol was detected using differential pulse voltammetry with a limit of detection as low as 40 ng mL^−1^ and linear ranges from 0.1 × 10^3^ to 3.6 × 10^3^ ng mL^−1^ and from 3.6 × 10^3^ to 1.5 × 10^4^ ng mL^−1^. The biosensor doesn’t exhibit sufficient sensitivity based on the MRL. The fabricated biosensor was used to determine CAP in spiked honey and milk samples with recovery percentages of 97–103%. An important advantage of the biosensor was the possibility of determination of CAP in food samples without any pre-treatment which is related to the performance of highly ordered cylindrical surfactant micelles.

Liu and co-workers developed an amperometric aptasensor for the detection of CAP. For construction of this aptasensor, a glassy carbon electrode surface was modified with a nanocomposite prepared from silver nanoparticles (AgNPs) and reduced graphene oxide (rGO) [60]. Then, CAP aptamers were immobilized on the surface of the modified electrode. Upon the binding interaction between CAP and its specific aptamer and then electrocatalytical reduction by the AgNPs/rGO nanocomposite, an electrochemical signal was produced. Due to the electrocatalytic activity of silver nanoparticles in CAP reduction, it was hybridized with rGO to produce a synergetic catalysis effect. Cyclic voltammetry (CV), and linear sweep voltammetry (LSV) were used for electrochemical measurements. Under optimized conditions, the aptasensor detected CAP with a detection limit of 0.65 ng mL^−1^ and a linear range of 3.23–1.13 × 10^4^ ng mL^−1^. Although compared to other studies, the LOD of this aptasensor is relatively good, it is lower than the MRL of CAP. The performance of the aptasensor was evaluated in spiked fresh milk and milk powder samples with a recovery percentages of 95–104%. The biosensor was selective and reproducible, but the incubation time with CAP was quite long (40 min).

Various analytical methods have been developed for simultaneous detection of multiple antibiotic residues. Among these, electrochemical biosensor have attracted considerable attention. Yan et al. described an electrochemical biosensor for simultaneous detection of chloramphenicol and oxytetracycline (OTC) using high-capacity magnetic hollow porous (MHP) nanotracer coupling exonuclease-assisted target recycling [61]. In this electrochemical aptasensor, MHPs were used for immobilization of metal ions (Cd^+2^ and Pb^+2^) in order to amplify the signal and simplify the separation and detection processes. Metal ion-immobilized MHPs were conjugated with DNA strands. For construction of the biosensor, complementary strands with CAP and OTC aptamers were immobilized on the surface of a glassy carbon electrode. In the absence of CAP and OTC, they formed a complex with a specific aptamer. After incubation with CAP and OTC, aptamers were released from double stranded DNA and conjugated MHPs were hybridized with the DNA strands on the surface of the electrode. Aptamer-CAP and aptamer-OTC were digested by exonuclease I and the target molecules released for another round of recycling, resulting in the signal improvement. Due to the dual signal amplification, detection limits of 0.15 and 0.10 ng mL^−1^ were obtained for CAP and OTC, respectively, which are satisfactory considering the MRL (0.3 ng mL^−1^ for CAP and 0.1 ng mL^−1^ for OTC), indicating the high sensitivity of the designed biosensor. The linear range between signals and the concentrations of CAP and OTC were obtained in the range of 5 × 10^−4^–50 ng mL^−1^. Cyclic voltammetry was used for electrochemical measurements. The aptasensor was evaluated with spiked milk samples and the recovery percentages were in the range of 94.9% and 104.2% for CAP, and 91.7% and 104.3% for OTC. The sensor has certain advantages such as selectivity and enough sensitivity for antibiotic detection in foods.

Another multiplex detection assay was reported by Chen et al. [62]. They developed an electrochemical aptasensor for multiplex detection of chloramphenicol and oxytetracycline using probe-based metal ions encoded with nanoscale metal-organic frameworks (NMOF) as a substrate, and circular strand-replacement DNA polymerization (CSRP) target triggered the amplification strategy. For construction of this biosensor, magnetic gold nanoparticles (MGNPs) were modified with assisted DNA (aDNA). Two captured DNA (cDNA) strands were used with their sequences composed of aptamer (specific for CAP and OTC) and the complementary sequence of the primer as a template for the polymerization reaction. Two reporter DNA (rDNA) strands were connected to metal ions encoded the NMOF. Hybridization of three strands (aDNA, cDNA and rDNA) with each other created a “Y-shape” structure. In the presence of target molecules (CAP and OTC), this Y-shape structure was dehybridized, due to aptamer binding to target, and rDNA released in the supernatant. The designed biosensor was highly sensitive (with respect to the MRL) with a detection limit of 0.1 × 10^−4^–0.2 × 10^−4^ ng mL^−1^ towards CAP and OTC, respectively, and a linear range between 0.3 × 10^−4^ and 16.1 ng mL^−1^. The high sensitivity of the biosensor was due to approximately 17 times amplification of the signal. The performance of the aptasensor was evaluated in spiked milk samples with a recovery percentage between 84.0% and 102.8%. A summary of reported studies on electrochemical biosensors for the detection of chloramphenicol is provided in Table 2.

### 3.3. Tetracycline

**Figure 5:**
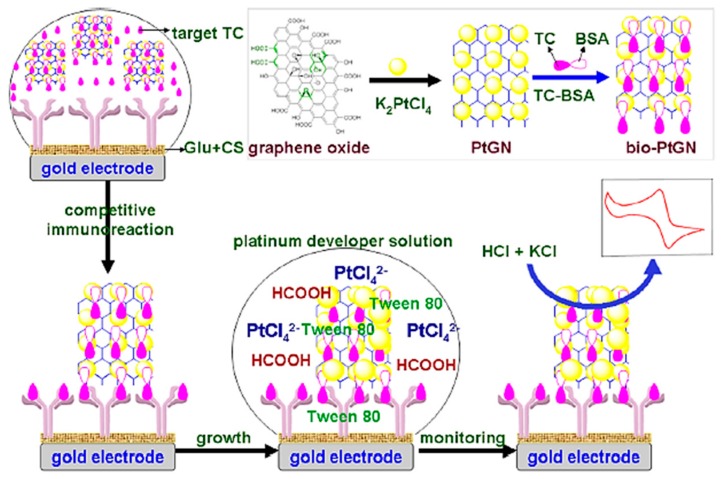
Schematic representation of electrochemical immunosensor based on platinum-catalyzed hydrogen evolution reaction for the detection of tetracycline (reprinted with permission from Que et al. [66]).

A variety of electrochemical biosensors for the detection of tetracycline (TC) have been designed. Que et al. [66] developed an electrochemical immunosensor based on a platinum-catalyzed hydrogen evolution reaction (HER) for the detection of tetracycline. For the construction of this immunosensor, graphene nanosheets (GN) were initially decorated by platinum nanoparticles (PtNPs). Then, the synthesized GN-PtNPs were used for the labeling of tetracycline-bovine serum albumin conjugates (TC-BSA, Figure 5). The assay was based on competitive binding of target tetracycline and TC-BSA labeled with GN-PtNPs to anti-TC antibody immobilized onto the surface of a gold electrode. The electrochemical signal was amplified via immersion of the immunosensor into a platinum developer solution containing [PtCl_4_]^2−^. The designed immunosensor was highly sensitive toward tetracycline with a detection limit of 6 pg mL^−1^ and a linear range of 0.05–100 ng mL^−1^. The immunosensor performance was validated with spiked milk, honey and peanut samples. The recovery percentage in these samples was in the range of 86–118%. According to the minimum level of spiking results (0.5 ng mL^−1^) and the MRL of TC in milk (100 ng mL^−1^), the immunosensor shows a high sensitivity. Moreover, the incubation time for TC detection was relatively short (30 min).

Aptamers are the most commonly employed bio-receptor elements in the electrochemical biosensors for the detection of tetracycline. An ultrasensitive M-shaped aptasensor was developed for quantitation of tetracyclines in food products [67]. In this electrochemical biosensor, potassium hexacyanoferrate (III) (K_3_[Fe(CN)_6_])/potassium hexacyanoferrate (II) trihydrate (K_4_[Fe(CN)_6_]·3H_2_O) solution was used as a redox probe for measurement of differential pulse voltammetry (DPV) and cyclic voltammetry (CV) output signal. 

**Figure 6:**
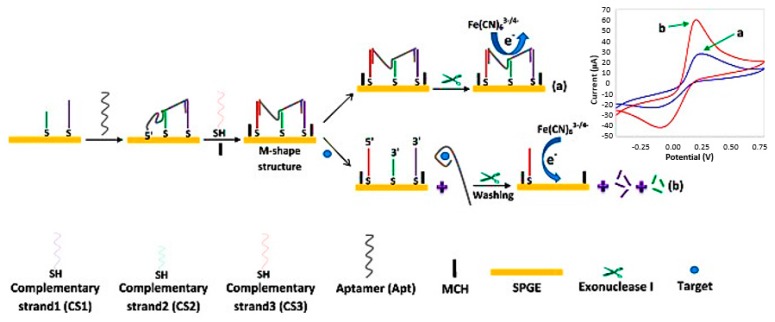
Assay formats of an electrochemical aptasensor for tetracycline detection. When tetracycline is absent, the M-shape structure is formed due to aptamer binding to CSs, resulting in the inhibition of redox probe access to the electrode surface and a reduced electrochemical signal (**a**). When tetracycline is present, it is captured by the aptamer and the M-shape structure is not formed. As a result of degradation of CS1 and CS2 by Exo 1, the redox probe has access to the electrode surface and the electrochemical signal increases (**b**). (Adapted from Taghdisi et al. [67] with permission).

First, three aptamer-complementary strands (CS1, CS2 and CS3) were immobilized onto the surface of screen-printed gold electrodes (SPGEs), followed by formation of an M-shaped structure due to binding of tetracycline aptamer (TC-apt) with three CSs. The complex of TC-apt-CSs decreased the access of redox probe to the electrode surface and led to a weak electrochemical signal. After incubation with tetracycline and its interaction with the aptamer, the M-shape structure was not formed and this led to an increased access of the redox probe to the electrode surface, and thus a strong electrochemical signal was obtained. The authors reached a limit of detection (LOD) as low as 0.19 ng mL^−1^ and a linear range of 0.67–1554 ng mL^−1^ (Figure 6). The designed biosensor was used to determine TC in spiked milk and serum samples. The recovery percentages in serum samples were 93.1% and 103.8%. The unique feature of the biosensor was its M-shape structure that made a significant difference in the peak current in the presence and absence of TC. The fabricated electrochemical aptasensor showed high selectivity toward TC with a detection time of 75 min. Sensitivity is an important factor in the design of any biosensor. A variety of strategies including application of nanomaterials have been employed to improve the sensitivity, especially where the detection of low level of analytes is highly desirable. The introduction of nanomaterials into electrochemical biosensors is considered as a novel approach for the construction of electrochemical biosensors. Nanomaterials have many advantages such as large surface area and high conductivity that make them a promising tools to modify the electrode surface in electrochemical biosensors. Carbon nanotubes are among the more attractive nanomaterials which are currently employed in electrochemical biosensors due to their unique electronic properties [68].

To obtain a highly sensitive electrochemical aptasensor for tetracycline, a glassy carbon electrode (GCE) was modified with multi-walled carbon nanotubes (MWCNTs) [69]. Firstly, carboxylated MWCNTs were coated onto the surface of the electrode, followed by covalent binding of amine-functionalized anti-TC aptamer. In this aptasensor, the MWCNTs were utilized as carriers for electron transfer between the ferricyanide (as redox probe) and the electrode surface to perform the electrochemical signal amplification. GCEs functionalized with MWCNTs/anti-TC aptamer presented a strong Faradaic current. The complex formation between aptamer and analyte (TC) increased the electron transfer resistance of [Fe_3_(CN)_6_]^3−^/[Fe_4_(CN)_6_]^2−^ redox probe and subsequently the generated current was decreased. It is worth mentioning that the sensitivity of the aptasensor increased significantly due to the increased conductivity. A detection limit of 2.22 ng mL^−1^ with a linear range of 4.44–2.22 × 10^4^ ng mL^−1^ was reported by the authors, which was satisfactory. Under optimized conditions, the TC detection time was 30 min. The aptasensor was employed for the detection of tetracycline in milk samples. The recovery percentage was in the range of 88–96%.

In addition to biological recognition elements such as antibodies and aptamers, researchers have recently introduced the utilization of synthetic materials in the fabrication of electrochemical biosensors. Molecular imprinted polymers (MIP) are among the promising artificial materials for the development of stable “solid-state like” artificial recognition elements. They are synthetic polymers that are produced by crosslinking monomers in the presence of a target analyte used as a template species. The use of MIP in the electrochemical biosensor for the tetracycline detection has been reported in the literature [70]. The biosensor was based on the electropolymerization of *p*-amino-thiophenol (PATP) along with functionalized gold nanoparticles on the surface of a gold electrode. Ferricyanide solution was used as a redox probe to perform the linear sweep voltammetric detection of tetracycline. The tetracycline binding to the specific cavity on the imprinted electrode increased the electron transfer with enhancement in the measured current. The MIP electrochemical biosensor was used for the detection of TC in spiked honey samples and the recovery percentages were in the range of 101.8–106.0%. A detection limit of 9.8 × 10^−8^ ng mL^−1^ with a linear range of 9.94 × 10^−5^–9.94 ng mL^−1^ was obtained with the purposed electrochemical artificial biosensor which indicates its high sensitivity. A significant increase in sensitivity was observed due to application of gold nanoparticles in the construction of the polymer matrix which were integrated to increase the conductivity of the transducer surface. In this research, an extraction time of 20 min and an incubation time of 30 min was chosen in order to obtain the optimal signal.

Magnetic nanoparticles (MNPs) are also considered as a good candidate in the development of electrochemical biosensors. This is mainly due to their particular characteristics such as large surface area, unique physicochemical properties and easy production. However, despite their numerous advantages, they are prone to aggregation, limiting their application in the construction of biosensors [71]. To overcome the abovementioned drawbacks, Zhan et al. [72] used MPNs (Fe_3_O_4_) along with reduced graphene oxide (rGO) and sodium alginate (SA) nanocomposite to construct a tetracycline electrochemical aptasensor. A screen printed carbon electrode was coated with rGO-Fe_3_O_4_/SA nanocomposite and TC-binding aptamer was subsequently immobilized on the modified surface. Tetracycline was quantified by differential pulse voltammetry with a detection limit of 0.27 ng mL^−1^ and a linear range of 0.44–2.22 × 10^3^ ng mL^−1^. The designed aptasensor provided a rapid, highly sensitive and selective platform for the detection of TC. The biosensor was not applied for the analysis of real food samples.

The oxidation properties of tetracycline have also been explored in the construction of TC electrochemical biosensors. Gan et al. [73] described a simple electrochemical biosensor based on the electrochemical oxidation of tetracycline. A mixture of Fe and Zn nanoparticles were incorporated into montmorillonite (MMT) nanolayers by cation-exchange method to enhance the MMT catalytic activity. Then, Fe/Zn-MMT as a sensing film was utilized for the surface modification of a glassy carbon electrode. The oxidation response of tetracycline on the surface of the fabricated electrode was studied using differential pulse voltammetry in the presence of sodium dodecyl sulfate (SDS) as an anodic reaction facilitator of TC. In the absence of tetracycline, the sensor showed no oxidation response. The designed sensor showed a detection limit of 4.44 ng mL^−1^ with a linear range of 133.2–23.088 × 10^3^ ng mL^−1^. Furthermore, the electrochemical biosensor was successfully employed for the detection of tetracycline in feedstuffs, chicken, fish and shrimp samples with recovery percentages of 97.2–103%.

A label-free electrochemical nanobiosensor was developed by Zhang et al. [74] for the detection of tetracycline. For the construction of the biosensor, a nanoporous silicon (PS) chip was firstly functionalized with amino groups through silanization in 3-aminopropyltriethoxysilane. Then, tetracycline aptamer was immobilized on the surface of the amino modified PS chip. Non-specific sites were blocked with bovine serum albumin (BSA). The fabricated biosensor was used for the determination of tetracycline based on electrochemical impedance spectroscopy (EIS). Tetracycline binding to the aptamer resulted in a decrease in impedimeric response. The nanobiosensor was able to detect tetracycline with a LOD of 0.89 ng mL^−1^ and a linear range from 0.93 to 27.71 ng mL^−1^. The biosensor was not applied for the analysis of real food samples or the evaluation of food matrix effects. 

Kim et al. [75] reported the development of an electrochemical aptasensor for the detection of tetracycline based on the immobilization of biotinylated TC aptamer on a streptavidin-modified screen-printed gold electrode. The binding of TC to aptamer was evaluated by cyclic voltammetry and square wave voltammetry (SWV) in the presence of ferricyanide as redox probe. Upon binding of tetracycline to the aptamer, the rate of electron flow of the redox probe was hindered, leading to a decreased electrochemical current. The presented sensing platform provided a higher sensitivity than a thiol-modified aptamer which can be attributed to the efficient attachment of biotinylated aptamer onto the surface of gold electrode. The designed aptasensor showed a detection limit of 4.44 ng mL^−1^ with a linear range of 4.44 ng mL^−1^–4.44 μg mL^−1^. The aptasensor was selective in a mixture of TC and other tetracycline derivatives (oxytetracycline and deoxycycline). The biosensor was not applied for the analysis of real food samples. Table 3 summarizes the analytical performance of the electrochemical biosensors for the detection of tetracycline reported in the literature.

### 3.4. Streptomycin

**Figure 7:**
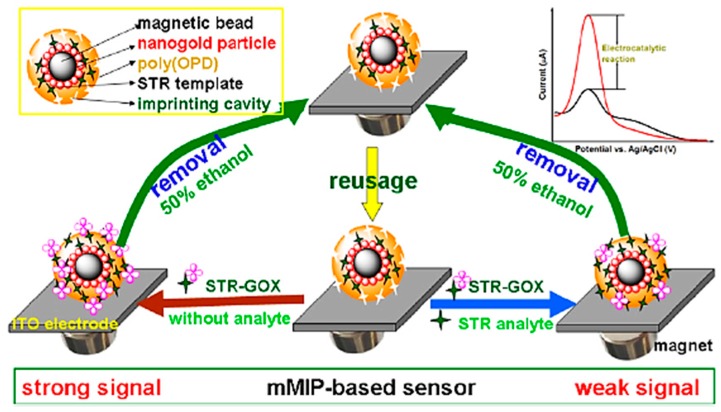
Schematic representation of Au(III)-promoted magnetic molecularly imprinted polymer nanospheres for the electrochemical detection of streptomycin (reproduced from Liu et al. [83] with permission).

Due to the potential risks of streptomycin, various methods have been recently developed for the detection of its residues in animal-derived foods. Among these methods, electrochemical biosensors have received particular attention. Que et al. [82] developed a molecularly imprinted polymer (MIP) for the electrochemical detection of streptomycin. The MIP was constructed by co-polymerization of aniline and *o*-phenylenediamine on the surface of a gold electrode. The sensor was based on the competitive binding of enzyme-labeled streptomycin and free streptomycin in the cavities on the MIP modified transducer surface. Catalytic oxidation of glucose by conjugated glucose oxidase generated the electrochemical signal. The method shows a high sensitivity due to its enzymatic amplification methodology. A detection limit of 7.0 pg mL^−1^ with a linear range from 0.01 to 10 ng mL^−1^ was obtained which shows the high sensitivity of the biosensor considering the MRL of streptomycin (200 ng mL^−1^ in milk). The biosensor was demonstrated in spiked milk and honey samples with recovery percentages from 82 to 124.24%. The biosensor was simple and selective towards other antibiotics. A similar approach was described by Liu et al. [83] using magnetic molecularly imprinted polymer nanospheres (mMIP). The mMIP nanospheres were synthesized using the assembly of [AuCl_4_]^−^ ions on the surface of magnetic beads (MBs) and then polymerization of *o*-phenylenediamine on the surface of functionalized MBs was carried out in the presence of STR templates. Au(III) ions caused the polymerization of *o*-phenylenediamine monomers onto the magnetic beads, while Au(III) ions were reduced to Au atoms. The prepared mMIP nanospheres were based on the competitive binding of target STR and STR labeled with glucose oxidase (GOx) to recognition cavity sites on the magnetic beads (Figure 7).

Catalytic oxidation of glucose substrate by GOx generated an amplified electrochemical signal. Under optimal conditions, a detection limit of 10 pg mL^−1^ with a linear range of 0.05–20 ng mL^−1^ was obtained. The sensor performance was successfully evaluated by spiked milk and honey samples with recovery percentages of 81–129%. The biosensor results for spiked samples showed good accordance with the results obtained by a high-performance liquid chromatography method. The reaction time for determination of STR was 10 min, which is excellent considering the high sensitivity of the biosensor.

In order to achieve a high sensitivity in electrochemical biosensors, nanoparticles can provide a good alternative to enzymes for signal amplification. Yin et al. [84] demonstrated an electrochemical aptsensor based on gold nanoparticle-functionalized magnetic multi-walled carbon nanotubes (GNP-MWCNTs-Fe_3_O_4_) and nanoporous PtTi (NP-PtTi) alloy for the detection of streptomycin. A bare glassy carbon electrode (GCE) surface was modified with GNP-MWCNTs-Fe_3_O_4_ composite followed by deposition of a NP-PtTi suspension. Then, STR aptamer was immobilized onto the NP-PtTi/GNP-MWCNTs-Fe_3_O_4_/GCE. Possible remaining active sites were blocked with bovine serum albumin (BSA) to avoid any nonspecific signals. MWCNTs-Fe_3_O_4_ nanocomposite was supposed to provide a large surface area and improved electronic conductivity. Furthermore, gold nanoparticles due to their unique properties such as large surface area, great electron transfer and biocompatibility could efficiently increase the immobilization content of STR aptamer. NP-PtTi alloy provided a matrix for effective immobilization of STR apatmer and a conductive pathway for electron transfer. Under optimized experimental conditions, the aptasensor showed an excellent sensitivity with a detection limit of 7.8 pg mL^−1^ and a linear range from 0.05–100 ng mL^−1^. The practical performance of the aptasensor was evaluated in spiked milk samples. The recovery percentage was in the range of 97.3–105.2%. The aptasensor showed a good reproducibility, high selectivity and stability. The incubation time for STR detection was 120 min, which is relatively long.

Yin et al. [85] also demonstrated an electrochemical biosensor based on “signal attenuation” for sensitive detection of streptomycin. In the given biosensor, electrochemical signal was amplified by utilization of nanomaterials including porous carbon nanorods (PCNRs), gold nanoparticles (AuNPs) and copper oxide (CuO) functionalized multiwalled carbon nanotube (MWCNTs) composites. A glassy carbon electrode was coated with porous carbon nanorods, and then MWCNTs-CuO-AuNPs suspension was subsequently casted onto the surface of the PCNRs-modified electrode. The resulting electrode was functionalized with streptomycin aptamer through interaction between the gold nanoparticles and the thiol groups of the aptamer. Complex formation between streptomycin and immobilized aptamer generated the attenuated output signal. Electrochemical measurements were performed in the form of differential pulse voltammetry (DPV). Under the optimized conditions, the reported apatasensor exhibited a detection limit of 0.036 ng mL^−1^ with a linear range of 0.05–300 ng mL^−1^. The aptasensor feasibility was demonstrated for the detection of streptomycin in milk and honey samples. The recovery percentage was from 96.7% to 105.8%. The detection time for STR was estimated at 120 min. The aptasensor exhibited an excellent selectivity and sufficient stability.

Danesh et al. [86] developed an electrochemical arch-shape aptasensor for quantitation of streptomycin in milk samples. The biosensor was based on a gold electrode, streptomycin aptamer, complimentary strand (CS) of aptamer, exonuclease I (Exo I) and ferricyanide as redox probe. STR aptamer was immobilized onto the surface of the gold electrode in an arch-shaped fashion with its complimentary strand. In the absence of STR, the arch-shape structure remained unchanged and CS was protected from Exo I. A relatively weak electrochemical signal was obtained due to the limited access of the redox probe to the electrode surface. After incubation with STR, CS was displaced with Exo I, and increased access of redox probe to the electrode surface led to a strong electrochemical signal. Differential pulse voltammetry (DPV) measurements of STR showed a detection limit of 6.62 ng mL^−1^ with a linear range of 17.43–871.5 ng mL^−1^. The designed electrochemical biosensor was used for STR measurement in spiked milk and serum samples which were complex biological fluids containing different components. Calculated LODs in spiked milk and serum samples were 11 ng mL^−1^ and 10.2 μg kg^−1^, respectively. The resulted LODs were higher than measured LOD in the buffer medium but were lower than the toxicity level of STR in blood (35 μg mL^−1^) and the maximum acceptable level of STR in milk (200 μg kg^−1^). The recovery percentages in serum samples were 95.4% and 98.2%.

**Figure 8:**
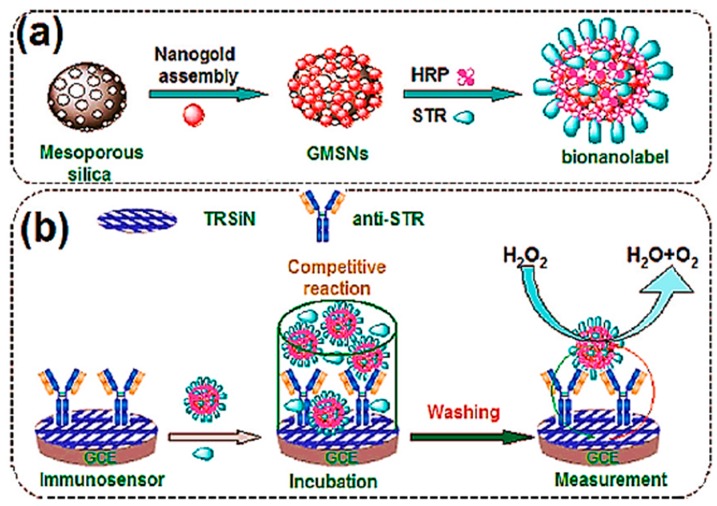
(**a**) Schematic illustration of the bionanolabel construction process and (**b**) fabrication process of the STR immunosensor and principle of the competitive assay (reproduced from Liu et al. [87] with permission from American Chemical Society).

In another study, Liu et al. [87] reported a simple electrochemical immunoassay for the sensitive detection of streptomycin. For the fabrication of the biosensor, monoclonal anti-STR antibodies were immobilized onto an organosilica colloid nanocomposite. Then, a glassy carbon electrode was coated on the sensing interface using a sol-gel method (Figure 8a). For the construction of bio-nanolabels, the mesoporous silica was firstly decorated with gold nanoparticles, and then horseradish peroxidase (HRP) enzyme and STR-bovine serum albumin (BSA) conjugates were co-immobilized on the transducer surface (Figure 8b). The streptomycin was measured based on the competition between STR analyte and STR-BSA conjugates. The assay was performed in the presence of hydrogen peroxide as enzyme substrate and electrochemical current was measured. In the presence of high concentrations of STR in the sample, the electrochemical current was decreased and vice versa. The required time for antigen-antibody interaction was 30 min which is satisfactory compared with other electrochemical biosensors. Under optimized conditions, the immunosensor showed a detection limit of 5 pg mL^−1^ with a linear range of 0.05–50 pg mL^−1^ which is excellent sensitivity. The immunosensor was employed to detect streptomycin in spiked milk, honey, kidney and muscle samples. The recovery percentage in spiked samples was 94–114%.

## 4. Conclusions and Future Prospects

From our overview of the monitoring of antimicrobial drug residues in animal-derived food, it can be concluded that nanomaterial-based electrochemical biosensors possess excellent value due to their rapid response, low cost and good selectivity and sensitivity, and hence have been extensively employed for the detection of antimicrobial drug residues. However, an important aspect in the construction of high-performance nanomaterial-based electrochemical biosensor platforms is the appropriate selection of transducer surface. It is very well established that good electrode materials have not only excellent conductivity, catalytic activity, and biocompatibility to accelerate signal transduction, but also amplify biorecognition events with specifically designed signal tags, resulting in high sensitivity. The incorporation of nanomaterials potentially increases the response speed, selectivity and sensitivity to meet the requirements of detection of contaminants in food samples. This review paper has highlighted the importance of diverse nanomaterials, such as metal nanomaterials, silica nanoparticles, carbon nanomaterials and other functionalized nanoparticles in the construction of electrochemical biosensors for monitoring antimicrobial drug residues in food samples. Despite of all this progress, future research must focus on various aspects to improve the monitoring of antimicrobial drug residues. For example, although electrochemical biosensors facilitated very low detection limits of kanamycin, tetracycline and streptomycin, however, a much needed improvement in the design of electrochemical biosensors is required to achieve the detection of chloramphenicol at low levels below the regulatory limits.

The design of array systems based on the immobilization of various biomolecules to identify different analytes simultaneously can reduce the analysis cost, and can be proved as a better detection platform for drug residues. The portability and reusability of electrochemical biosensors can be another attractive feature for the future research to meet the needs for rapid, on-site detection. Lastly, a new trend can be use of nanolabels to replace the enzyme labels in the affinity-based electrochemical biosensors to perform highly sensitive detection of antimicrobial drug residues in food samples.

## Figures and Tables

**Table 1:** Electrochemical biosensors for the determination of kanamycin in food samples.

Serial Number	Assay/Principle	LOD * (ng/mL)	Linear Range (ng/mL)	Sample	Reference
1	Amperometric immunosensor	0.00574	0.01–12	Food	[36]
2	Square wave voltammetry based immunosensor	0.015	0.050–16	Pork meat	[37]
3	Square wave voltammetry based immunosensor	0.00631	0.02–14	Food	[45]
4	Square wave voltammetry based aptasensor	6.783	4845–9.69 × 10^6^	Milk	[46]
5	Photoelectrochemical aptasensor	96.9	484.5–111,435	-	[47]
6	Differential pulse voltammetry based aptasensor	0.0037	0.05–100	Milk	[48]
7	Differential pulse voltammetry based aptasensor	0.00042	50 × 10^−7^–50 × 10^−2^	Food	[49]
8	Differential pulse voltammetry based aptasensor	2810	1 × 10^−8^–1.5 × 10^−7^	Milk	[34]
9	Differential pulse voltammetry based aptasensor	8.6	0.01–200	Milk	[50]
10	Differential pulse voltammetry based aptasensor	46 × 10^−6^	50× 10^−6^–40 × 10^−2^	Food	[51]
11	Electrochemical impedance spectroscopy based aptasensor	0.11	1.2–75	Milk	[44]

* The LOD was determined in buffer medium.

**Table 2:** Summary of reported studies on electrochemical biosensors for the detection of chloramphenicol.

Serial Number	Assay Format	LOD * (ng/mL)	Linear Range (ng/mL)	Sample	Reference
1	Differential pulse voltammetry based molecularly imprinted sensor	0.032	1.615–161.5 and 161.5–1292	Milk and honey	[52]
2	Differential pulse voltammetry based aptasensor	0.059	0.097–0.626	Milk	[53]
3	Differential pulse voltammetry based immunosensor	0.06	0.1–1000	Beef, fish, pork	[54]
4	Differential pulse voltammetry based aptasensor	0.094	0.323–323	Honey	[55]
5	Differential pulse voltammetry sensor	48.45	323–3.65 × 10^4^	Milk and eye drops	[57]
6	Differential pulse voltammetry sensor	20.99	32.3–3.23× 10^5^	Eye drops	[58]
7	Differential pulse voltammetry sensor	40	0.1 × 10^3^–3.6 × 10^3^ and 3.6 × 10^3^–1.5 × 10^4^	Milk and honey	[59]
8	Cyclic voltammetry and linear sweep voltammetry based aptasensor	0.65	3.23–1.13 × 10^4^	Fresh milk and milk powder	[60]
9	Cyclic voltammetry based aptasensor	0.15	5 × 10^−4^–50	Milk	[61]
10	Square wave voltammetry based aptasensor	0.1 × 10^−4^	0.3 × 10^−4^–16.1	Milk	[62]
11	Differential pulse voltammetry based immunosensor	0.11	0.2–80.0	Milk	[63]
12	Amperometric sensor	1.615	323–1938	Milk	[64]
13	Potentiometry based molecularly imprinted sensor	323	323–3.23 × 10^6^	Pharmaceutical drugs	[65]

* The LOD was determined in buffer medium.

**Table 3:** Summary of reviewed studies on electrochemical biosensors for the detection of tetracycline.

Serial Number	Assay Format	LOD * (ng/mL)	Linear Range (ng/mL)	Sample	Reference
1	Cyclic voltammetry based immunosensor	0.006	0.05–100	Milk, honey and peanut	[66]
2	Differential pulse voltammetry (DPV) based aptasensor	0.19	0.67–1554	Milk	[67]
3	Differential pulse voltammetry based aptasensor	2.22	4.44–2.22 × 10^4^	Milk	[69]
4	Linear sweep voltammetry based molecularly imprinted sensor	9.8 × 10^−8^	9.94 × 10^−5^–9.94	Honey	[70]
5	Differential pulse voltammetry based aptasensor	0.27	0.44–2.22 × 10^3^	-	[72]
6	Differential pulse voltammetry based sensor	4.44	133.2–23.088 × 10^3^	Feedstuff, chicken, fish and shrimp	[73]
7	Electrochemical impedance spectroscopy based aptasensor	0.89	0.93–27.71	-	[74]
8	Cyclic voltammetry and square wave voltammetry (SWV) based aptasensor	4.44	4.44–4.44 × 10^3^	-	[75]
9	Electrochemical impedance spectroscopy (EIS) based aptasensor	0.001	0.005–5.0	Milk	[35]
10	Cyclic voltammetry based molecularly imprinted sensor	0.04	0.1–40	-	[76]
11	Cyclic voltammetry based aptasensor	1	0.1–100	Milk	[77]
12	Amperometry based immunosensor	0.86	2.84–171	Milk	[78]
13	Electrochemical impendence spectroscopy (EIS) based aptasensor	10	10–3.0 × 10^3^	Milk	[79]
14	Differential pulse voltammetry based aptasensor	0.25 × 10^−2^	0.04–4.44 × 10^5^	Milk	[80]
15	Cyclic voltammetry sensor	0.09	1.0–10.0	Water	[81]

* The LOD was determined in buffer medium.
